# Using fMRI to Investigate Memory in Young Children Born Small for Gestational Age

**DOI:** 10.1371/journal.pone.0129721

**Published:** 2015-07-01

**Authors:** Henrica M. A. de Bie, Michiel B. de Ruiter, Mieke Ouwendijk, Kim J. Oostrom, Marko Wilke, Maria Boersma, Dick J. Veltman, Henriette A. Delemarre-van de Waal

**Affiliations:** 1 Department of Pediatrics, VU University Medical Center, Amsterdam, The Netherlands; 2 Department of Psychosocial Research and Epidemiology, Netherlands Cancer Institute, Amsterdam, The Netherlands; 3 Department of Pediatric Psychology, VU University Medical Center, Amsterdam, The Netherlands; 4 Department of Pediatric Neurology and Developmental Medicine and Experimental Pediatric Neuroimaging Neuroimaging Group, Children’s Hospital, University of Tübingen, Tübingen, Germany; 5 Department of Clinical Neurophysiology, VU University Medical Center, Amsterdam, The Netherlands; 6 Department of Psychiatry, VU University Medical Center, Amsterdam, The Netherlands; 7 Department of Pediatrics, Leiden University Medical Center, Leiden, The Netherlands; Massachusetts General Hospital, UNITED STATES

## Abstract

**Objectives:**

Intrauterine growth restriction (IUGR) can lead to infants being born small for gestational age (SGA). SGA is associated with differences in brain anatomy and impaired cognition. We investigated learning and memory in children born SGA using neuropsychological testing and functional Magnetic Resonance Imaging (fMRI).

**Study Design:**

18 children born appropriate for gestational age (AGA) and 34 SGA born children (18 with and 16 without postnatal catch-up growth) participated in this study. All children were between 4 and 7 years old. Cognitive functioning was assessed by IQ and memory testing (Digit/Word Span and Location Learning). A newly developed fMRI picture encoding task was completed by all children in order to assess brain regions involved in memory processes.

**Results:**

Neuropsychological testing demonstrated that SGA children had IQ’s within the normal range but lower than in AGA and poorer performances across measures of memory. Using fMRI, we observed memory related activity in posterior parahippocampal gyrus as well as the hippocampus proper. Additionally, activation was seen bilaterally in the prefrontal gyrus. Children born SGA showed less activation in the left parahippocampal region compared to AGA.

**Conclusions:**

This is the first fMRI study demonstrating different brain activation patterns in 4-7 year old children born SGA, suggesting that intrauterine growth restriction continues to affect neural functioning in children later-on.

## Introduction

In children who are born small for gestational age (SGA), a suboptimal intrauterine environment has led to underdevelopment of both the body and the brain [1–4]. Suboptimal intrauterine environment may result from placental insufficiency, the most common cause of intra-uterine growth restriction (IUGR) [3]. SGA is associated with increased neonatal morbidity and mortality, short stature and metabolic disturbances [1], and is characterized by decreased body length and/ or weight and a diminished head circumference at birth. In the majority of these children, the delay in body growth is spontaneously restored during the first two years of life (SGA+) [2]. Approximately 10% lack catch-up growth and exhibit persistent short stature (SGA-). Failure of catch-up growth is associated with IUGR severity [5]. In addition to a negative influence on physical parameters, decreased intelligence levels and impaired cognition have been described in SGA children [6,7]. This is exemplified by SGA- children having a poorer school performance and experiencing more learning difficulties compared to healthy children [8,9]. Interestingly, SGA+ is associated with relatively better cognitive outcomes [6,10,11].

Animal studies demonstrate histopathological changes in the brains of SGA animals showing that IUGR affects brain development [12]. In recent years, human studies on brain anatomy in SGA individuals have used ultrasound and several MRI modalities to demonstrate differences in brain structure in SGA individuals. In line with animal studies, human studies show structural changes throughout the brains of SGA individuals both prenatally and at term age [6,13–15] in early life [16,17] extending into adulthood [18]. Studies demonstrate smaller total brain volumes, lower volumes of cortical grey matter in frontal, parietal, temporal, insular [19] and hippocampal regions and decreased white matter volumes. In addition, evidence of delayed cortical development [14,15], and altered brain network topology has been recently reported in young SGA children [15,20].

Functional MRI (fMRI) allows to study brain function, by assessing neuronal activity through changes in blood oxygenation levels. To our knowledge, no fMRI studies have been performed in young SGA children.

In the present study, we assess learning and memory in a sample of SGA children and focus on the medial temporal lobe (MTL) because of its key role in learning and memory [10,21]. We developed an fMRI encoding task involving picture encoding and postponed recognition [7,8,10], suitable for children in the age range of 4–7 years. Functional neuroimaging studies have demonstrated that the hippocampus and surrounding perirhinal and parahippocampal cortices, as well as prefrontal cortex (PFC) brain regions are active during episodic encoding in both adults and children [7,9–11,22–24].

The aim of our study was to investigate memory performance and patterns of neural activation in SGA children relative to children born with an appropriate weight for their gestational age (AGA). The effect of catch-up growth was studied by comparing a group of SGA children showing catch-up growth to a group of SGA children with persistent short stature.

## Methods

### Participants

The present study is part of a longitudinal study on brain development and cognition in children born SGA before and during growth hormone treatment (Dutch Trial Register: NTR 865), and describes baseline data. Following the International Small for Gestational Advisory Consensus Board Development Conference Statement [25], SGA was defined as a birth weight and/ or birth length ≤ -2 standard deviations (SD), adjusted for gender and gestational age; SGA+ was defined as postnatal catch-up growth with an actual height of less than 2 SD below the Dutch population reference mean and SGA- as persistent postnatal growth failure based on an actual height of less than 2 SD below this mean [26]. Parents of sixty five children gave written consent and could be included. Ultimately, data from 52 children could be used for analysis (for exclusion details, see Results section). For optimal comparison, a healthy control group of AGA children was selected to control for effects of age, gender and gestational age. Children were between 4 and 7 years old at the time of the study. Eighteen children were born AGA and 34 were born SGA of which 18 displayed postnatal catch-up growth (SGA+) and 16 children had persistent short stature (SGA-) (Table 1). SGA children were selected from the pediatric departments of the VU University Medical Center or one of the other participating hospitals in The Netherlands. Exclusion criteria were 1) prematurity below 34 weeks, 2) multiple birth, 3) impaired perinatal adaptation, indicated by an Apgar score < 7 after 5 min, 4) growth failure due to other somatic or chromosomal disorders or syndromes, 5) previous or present use of medication that could interfere with growth, and 6) learning difficulty (defined as estimated IQ-scores below 70) [27]. Parental educational levels were assessed according to the International Standard Classification of Education 1997. Due to recent concerns regarding the effects of anaesthetics on post-natal brain development [28,29], the number of procedures under general anaesthesia was also documented. The study was approved by the ethics committee of the VU University Medical Center, Amsterdam, The Netherlands. Each child gave verbal assent and written informed consent was obtained from the parents or guardians of each child according to the Declaration of Helsinki [30] prior to participation.

**Table 1:** Characteristics of study groups (N = 52 children).

		SGA			Main effect subgroup analysis (AGA vs SGA+ vs SGA-)	
	AGA(n = 18)	SGA+ (n = 18)	SGA–(n = 16)	SGA total group (n = 34)	F value	p Value
Gender (boys:girls)	9:9	10:8	9:7	19:15		ns
Handedness (right:left)	16:2	17:1	14:2	31:3		ns
Gestational age in weeks	39.4 (2.1)	38.8 (1.8)	39.2 (2.0)	39.0 (1.9)	0.3	0.7
Birth weight in grams	3504 (609)	2245 (324)	2447 (446)	2340 (394)	36.0	<0.001
Birth weight SD	0.3 (0.9)	-2.6 (0.3)	-2.3 (0.4)	-2.5 (0.4)	114.4	<0.001
Length SD at birth^a^	0.5 (0.9)	-1.5 (0.9)	-2.5 (0.8)	-2.1 (0.9)	57.2	<0.001
Head circumference SD at birth	0.1 (0.8)	-1.1 (0.7)	-0.9 (1.1)	-1.0 (0.9)	8.7	0.001
Age at MRI investigation in years	6.3 (1.0)	5.9 (0.9)	5.8 (1.1)	5.9 (1.0)	1.0	0.4
Length at MRI investigation in cm	120.6 (9.5)	117.5 96.3)	103.0 (6.0)	110.7 (9.5)	26.3	<0.001
Length SD at MRI investigation	0.0 (0.9)	-0.2 (0.7)	-2.9 (0.4)	-1.5 (1.5)	86.1	<0.001
Weight at MRI investigation in kilograms	23.2 (6.1)	20.4 (3.9)	14.8 (1.6)	17.6 (4.1)	16.0	<0.001
Weight SD at MRI investigation^b^	-0.1 (0.7)	-0.7 (1.2)	-1.4 (0.9)	-1.0 (1.0)	7.8	0.001
Head circumference at MRI investigation in cm	52.2 (1.7)	51.2 (1.1)	49.0 (1.6)	50.3 (1.7)	18.8	0.001
Head circumference SD at MRI investigation	0.5 (0.8)	-0.1 (0.6)	-1.3 (1.0)	-0.6 (1.0)	22.7	<0.001
Parental education						
≥ upper-secondary education (fathers) in %	94	76	87	81		ns
≥ upper-secondary education (mothers) in %	94	76	93	84		ns

Note. Data (except gender and handedness) are presented as mean (± standard deviation); p-value< 0.05 is considered significant, p-values between 0.05 and 0.10 are reported. AGA = appropriate for gestational age; SGA+ = small for gestational age with postnatal catch up growth; SGA– = small for gestational age without postnatal catch up growth; MRI = magnetic resonance imaging; SD = standard deviation; ns = non significant.

^a^When birth length was not available, measure of body length at first visit at child health centre was taken

^b^weight for length SD

### Experimental procedures

Before testing, parents of all participating children received a DVD with instructions on experimental procedures. The DVD contained a dummy version of the memory fMRI task, allowing the child to practice at home in order to familiarize him/herself with the task concept. Two pictures (bird and shoe) of this dummy version were used later on in the picture encoding and recognition task to establish a functional baseline of overlearned pictures. Practice time at home was not monitored. Test administration was spread over two days. On the first day, children underwent neuropsychological testing, using a personal desktop computer. Hereafter, they were prepared according to our mock scanner training protocol [31]. Children who failed this MRI training protocol were excluded since sedation was not applied. Handedness was assessed by observation which was confirmed by the child and/or parent. Also, familiarity with the fMRI task and procedure was checked for each child. The actual MRI procedure was conducted on the second day and lasted approximately 30 minutes. fMRI scans were acquired before structural MRI scans. Children performed the recognition part of the fMRI task outside the scanner, approximately 20 minutes after having completed the fMRI encoding task in the scanner.

### Neuropsychological assessment

#### Intelligence

Intelligence quotients (IQ) were estimated on the basis of a four-subtest short form of the Wechsler’s scales, yielding an estimate of the Full Scale IQ (eIQ) that would ordinarily be obtained by administration of the complete scales. Estimates of reliability and validity indicate the abbreviated forms of the Wechsler Preschool and Primary Scale Intelligence–Revised (WPPSI-R, Dutch version), for children under 6 years and the Wechsler Intelligence Scale for Children–third Edition (WISC-III, Dutch version) for children 6 years and older to approximate the Full Scale IQ when time limitations are a consideration [32,33]. The short form of the WPPSI-R consisted of the subtests Block Design, Arithmetic, Vocabulary, and Picture Completion. The short form of the WISC-III consisted of the subtests Block Design, Picture Arrangement, Similarities, and Vocabulary.

#### Learning and memory

Learning Locations (computerized non-verbal memory task) [34] requires the children to memorise the locations of 16 coloured pictures of natural objects. Dependent variables were total immediate recall, proactive interference, retroactive interference, delayed recall, delayed recognition and response times. Total immediate recall scores were calculated based on the sum scores of five identical memory trials, with a maximum score of 5x16 = 80. Proactive interference score was defined as the number of correctly recalled locations after rearrangement of original location as learned in the first five memory trails (maximum = 16). Retroactive interference scores indicated correctly recalled locations as learned in the first five identical memory trails, after the proactive interference phase, corrected for recall trail 5 ((recall memory trail 5 minus retroactive interference trail)/ recall trail 5). Delayed recall scores indicated the number of correctly recalled locations, 30 minutes after learning the five identical memory trails, corrected for recall trail 5 ((recall memory trail 5 minus delayed recall)/ recall trail 5). Delayed recognition scores were calculated based on correct identification whether or not a picture was presented at the original location, learned in the first five memory trails (maximum = 16).

The ‘Digit Span’ of the Dutch WISC-III was administered in children 6 years and older. As digits have poor relevance for younger children, nouns with an imaginable theme were used in 4 and 5 year-olds. Dependent variables were: (1) Span forwards: number of digits/ nouns in the longest string that the child repeated correctly in forward order (maximum = 9); (2) Span backwards: number of digits/ nouns in the longest string that the child repeated correctly in backward order (maximum = 8).

### Picture encoding & recognition task

#### Encoding phase

**Fig 1:**
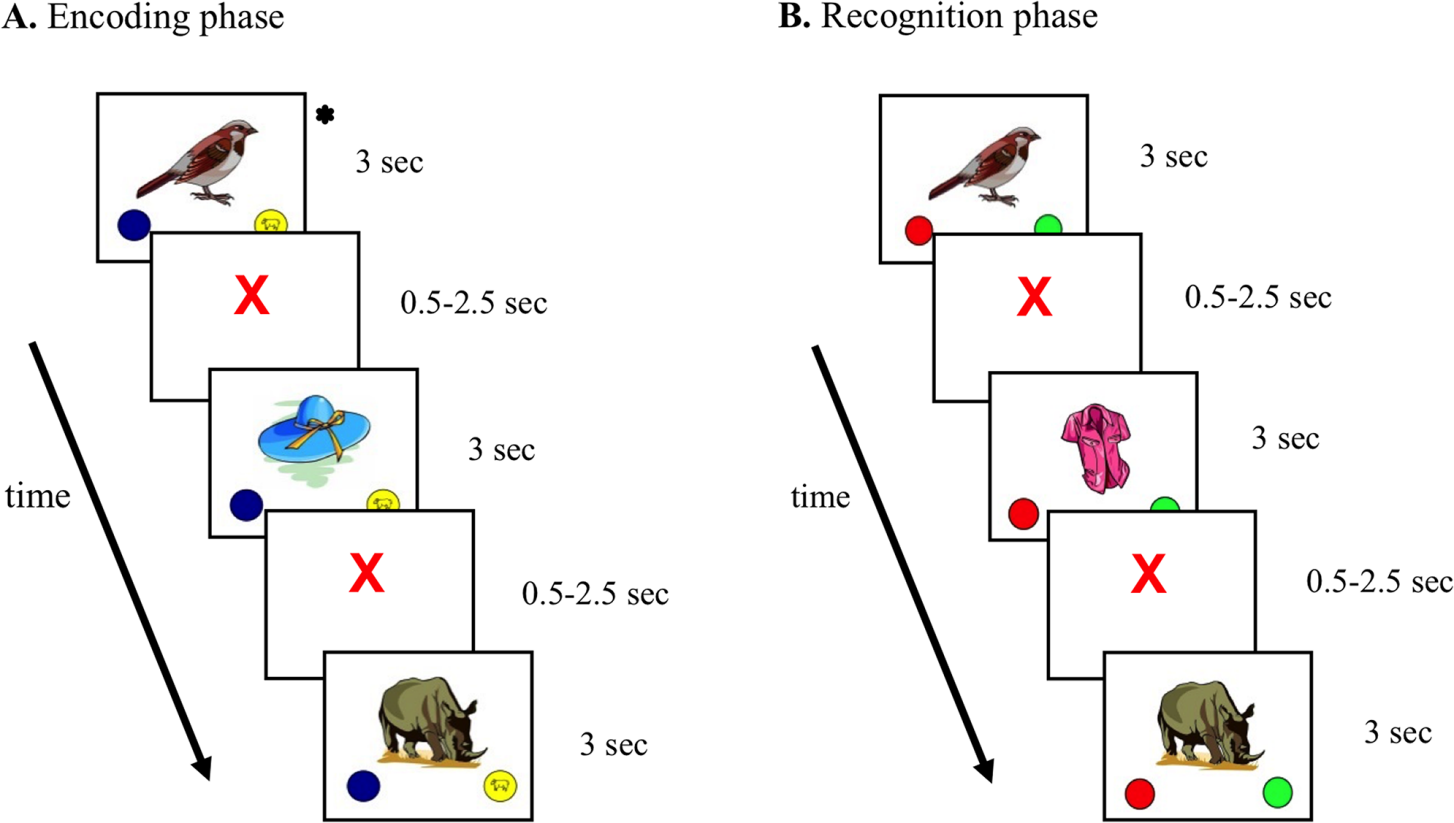
Design of picture encoding and memory task. (A) The encoding phase consisted of two runs with 30 novel and two recurring overlearned pictures (bird and shoe; presented 13 times), derived from the dummy memory disc which the child practiced at home. Children had to indicate whether the stimuli was an animal or object, while fMRI was administrated. (B) The recognition phase, administrated on a personal desktop computer, presented two times 30 pictures earlier presented in the encoding phase, 26 times two overlearned pictures (bird and shoe) and 60 novel pictures. Children had to decide whether or not the stimuli was earlier presented during the encoding phase. In both phases pictures were presented in random order during 3.0 seconds each, followed by a randomized interstimulus interval of 0.5–2.5 seconds during which a red fixation cross was presented. * Overlearned picture (bird or shoe), used to establish a functional baseline (fMRI)

The fMRI task was a non-verbal picture encoding task (Fig 1A), exclusively designed for this study. Stimuli were full colour cartoon pictures of objects and animals (www.clipart.com), previously tested with children to ensure familiarity [35]. Pictures were presented using Eprime software (Psychology Software Tools, Inc., Sharpsburg, PA, USA) and were projected on a white screen positioned in front of the scanner that could be seen through a mirror attached to the head coil. Children lay supine in the scanner while head movement was limited by foam padding within the head coil. Before presenting any stimuli, children were explicitly instructed to memorize the presented pictures for a later memory check. The encoding phase consisted of two runs of 30 novel (target pictures) and 13 presentations of two overlearned pictures derived from the dummy memory task (bird and shoe), counterbalanced across children. All pictures were presented in a recurrent and random order during 3.0 seconds each, followed by a randomized interstimulus interval of 0.5–2.5 seconds during which a red fixation cross was presented, centrally located on the white screen. To monitor task attention, children were requested to perform a semantic judgment (perceptual identification) for each drawing (animal vs object). They were asked to push any key of a MRI-compatible 4-key yellow response button box (Lumitouch, Photon Control Inc, Baxter, Canada) attached to the right hand when an animal was presented, and any key of a blue button box attached to the left hand in case of an object. Subject's performance (accuracy, defined as proportion of correct responses) and response times (RT) were administered electronically through the button boxes. The duration of the encoding phase was 7 minutes.

#### Recognition phase

During the recognition phase following fMRI data acquisition (Fig 1B), a total of 146 stimuli were presented on a personal desktop computer outside the MRI-scanner room. The stimuli concerned 2x30 target pictures presented during the encoding phase, 26 presentations of the 2 overlearned pictures of the dummy memory task also presented during the encoding phase (bird and shoe), and 60 novel pictures. All pictures were presented in random order during maximally 3.0 seconds each, followed by a randomized interstimulus interval of 0.5–2.5 seconds during which a red fixation cross was presented, centrally located on the white computer screen. Children were requested to press a green button on a keyboard if they recalled a picture as ‘familiar’ (registered as ‘presented’ during the fMRI encoding task) and a red button if they judged a picture as ‘unfamiliar’ (registered as ‘not presented’ during the fMRI task). ‘Sensitivity probability’ (Pr) (proportion of hits—proportion of false alarms (FA)) and ‘response bias’ (Br) (FA/[1 –Pr]) were calculated on the basis of proportions of hits (familiar pictures, known from the encoding phase, correctly judged ‘familiar’) and false alarms (FA)(unfamiliar pictures incorrectly judged as ‘familiar’), Recognition data were lost for four subjects due to equipment failure. The duration of the recognition phase was approximately 10 minutes.

### Image acquisition and preprocessing

MRI data was collected at the VU University Medical Center using a 1.5-T Sonata scanner (Siemens, Erlangen, Germany) with an 8 channel head coil array. T2*-weighted echoplanar images sensitive to blood oxygenation level dependent (BOLD) contrast covering the whole brain (35 coronal slices of 2.5mm thickness with an in-plane resolution of 3×3mm; TR 2.25s, TE 45ms, flip angle 90^0^) were acquired. In addition, a high-resolution T1-weighted scan using a 3D Magnetization Prepared Rapid Gradient Echo (MPRAGE) sequence was acquired for each participant for coregistration with the fMRI data [36]. The sequence parameters of the T1-weighted images were TR 2700 ms; TE 3.97ms; voxel size 1.0*1.0*1.5mm; flip angle 8°; 160 coronal slices; FOV of 250mm covering whole brain; total acquisition duration: 4:53 minutes. Preprocessing and data analysis were performed using Statistical Parametric Mapping software (SPM5; Wellcome Department of Cognitive Neurology, London, UK) implemented in Matlab (The Mathworks, Natick, MA). The first two scans (acquired during initial presentation of fixation cross) were discarded to allow for steady-state magnetization. Images were realigned and unwarped, correcting for motion and motion*B0 interaction [37] and corrected for slice acquisition delays. Next, T1-coregistered volumes were spatially normalized to a custom-generated pediatric template [38] using unified segmentation [39]; the thus-generated spatial normalization parameters were then applied to the functional data, which was finally spatially smoothed using an 8-mm full-width at half maximum Gaussian kernel. Total displacement and scan to scan displacement was assessed for each subject by taking both translation and rotation (obtained from the realignment parameters) into account [40,41]. They describe overall subject motion relative to the first scan and relative to the previous volume, respectively. When total displacement exceeded 4mm, the dataset was excluded.

### Statistical analysis

For fMRI data of each subject, trial-related activity was analysed with the general linear model (GLM) in SPM (event-related-design). The fMRI time series data were modelled using delta functions convolved with a canonical haemodynamic response function (HRF) to study the effects of interest, generating parameter estimates for novel trials and overlearned trials (control condition). Activation difference between the encoding of novel and overlearned pictures were calculated in Z scores. Z scores are standardized statistical values with higher scores corresponding to lower p-values. For instance, a Z score of 3.08 corresponds to a p value of .001 Realignment parameters were included as regressors of no interest. Statistical parametric maps were created for each subject by applying linear contrasts to the parameter estimates for these events of interest. Contrast images from the individual, first level were entered into a second-level (random-effects) analysis to assess group effects. Group comparison focussed on the novel vs. overlearned (control condition) effect and was performed with and without age and IQ as covariates. Main effects for each group are reported at p<0.05 corrected for multiple comparisons using the false discovery rate (FDR) [42]. Interaction effects are reported at p<0.001 uncorrected, masked with the orthogonal main effect at p< 0.05, equivalent Z>3.89. For our a priori regions of interest (ROI), left and right parahippocampal gyrus and hippocampus proper, small volume correction was applied by centering a 10 ml (13.4 mm radius) sphere around the peak voxel. The resulting volumes of interest had to meet p<0.05 FDR corrected to be considered significant [43].

Statistical analyses, other than those included in SPM were performed using SPSS version 16.0 (Chicago, IL, USA). Analysis of baseline characteristics was performed using analysis of variance (ANOVA). Chi-square test was used for categorical baseline characteristics (sex, handedness and parental educational levels). Group comparisons of fMRI performance and neuropsychological data were performed using a general linear model (GLM) analysis (multivariate, full factorial, difference contrast) with subject group (AGA and SGA total [combined SGA- and SGA+] and subgroups [AGA, SGA+ and SGA-]) as fixed factor and age and response time (when applicable) as covariate. Subsequently, to investigate a trend between the three subgroups (AGA vs. SGA+ vs. SGA-) a linear polynomial contrasts analysis was included in the GLM. As motion parameters cannot be expected to be normally distributed, a Mann-Whitney-u-Test was used to assess differences between groups. Two-tailed P-values < 0.05 were considered significant. For multiple comparisons, a Bonferroni-corrected level of significance was applied.

## Results

### Participants

Sixty-five children underwent the mock scanner training protocol. Seven children (11%, mean age 4.7 years; range 4.2–5.4 years, six boys vs one girl) were not able to finish the mock scanner training due to anxiety. The remainder fifty-eight (89%) children passed the training, underwent the MRI procedure and completed the MRI session, including fMRI-scanning. One boy was excluded because of a later established IGF1-R mutation; four children were excluded due to excessive motion (range 5-13mm, mean age 5.6 years; range 4.8–6.8 years) during fMRI scanning. Finally, one child was excluded due to equipment failure during scanning. Three children (1 AGA, 2 SGA+) underwent a single short anaesthetic procedure because of a simple ear-nose-throat procedure ((adeno)tonsillectomy). None of the other children have been on anesthesia ever. Since it only concerned 3 children, we did not account for anaesthesia in our analyses as additional variable. The characteristics of the remaining 52 (80%) children in the AGA, SGA+ and SGA- groups are listed in Table 1. Characteristics of the excluded children were similar to the 52 included children. As expected, birth weight and head circumference at birth were lower in both SGA groups compared to AGA. The SGA- and SGA+ group did not differ with respect to birth weight and head circumference at birth. Body length and head circumference at the time of MRI investigation was significantly lower in the SGA- group when compared with either the SGA+ or AGA group. With respect to motion characteristics, 9 out of 52 children had a maximum displacement between 3 and 4mm and motion, which was similar in each subgroup (F(2,49) = 1.09, p = .345). Right and left handedness percentages were equal in all groups. The percentage of parents reaching ≥ post-secondary education was relatively high in AGA and SGA- vs SGA+ group, and reached level of significance with respect to AGA compared to SGA+ (F_father_(2,46) = 4.57, p = .015; F_mother_(2,46) = 4.81, p = .021).

### Neuropsychological testing

**Fig 2:**
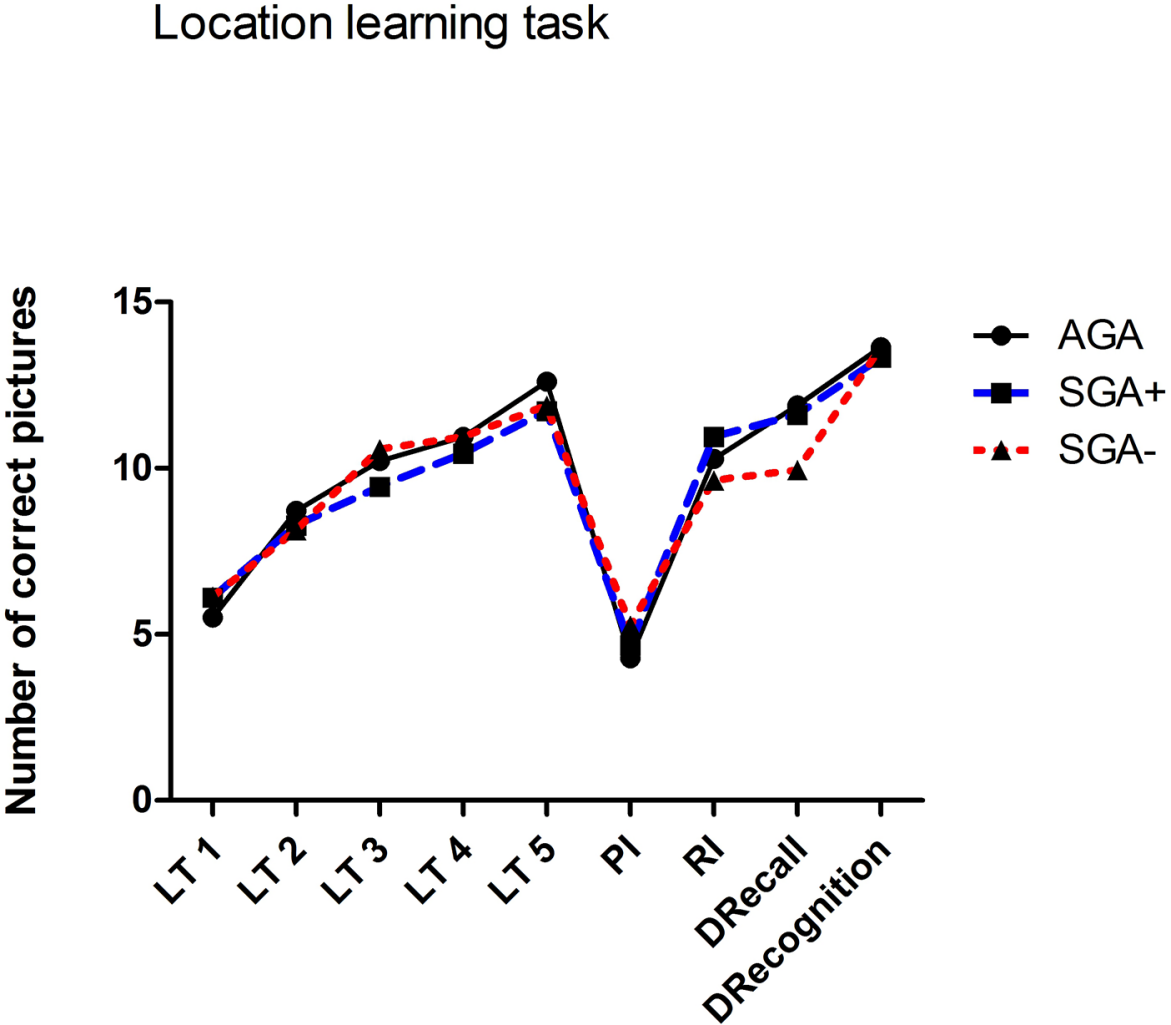
Location learning task. Mean raw scores for group of AGA, SGA+ and SGA- children. LT1 –LT5 = direct recall trail scores, PI = proactive interference score, RI = retroactive interference score, DRecall = delayed recall scores (30 minutes after direct recall trail 5), DRecognition = delayed recognition, AGA = average for gestational age, SGA+ = small for gestational age with catch-up growth, SGA- = small for gestational age without catch-up growth.

None of the children scored below 70 (not even below 80) on the short form Wechsler IQ-tests. Although within the normal range, children born SGA showed lower IQ scores compared to AGA children (Table 2). Subgroup comparison showed that this difference was driven mainly by the SGA- children. Difference between SGA+ group and SGA- group did not reach significance (p = .561). Linear polynomial contrast analysis showed a significant trend for IQ over the three groups (p = .005), with lowest IQs in SGA- children. Compared to AGA children, the word/ digit span forwards of children born SGA was significantly poorer and persisted after covarying for IQ. Word/ digit span performance of SGA+ and SGA- children was similar. Although AGA children performed better on word/ digit span backwards than SGA children, this effect disappeared after covarying for IQ. In general, performances of location learning task were similar in all three subgroups after covarying for corresponding response times (Fig 2).

**Table 2:** Intelligence, word/ digit spans and location learning (raw scores) of children born AGA, SGA+ and SGA–(N = 52).

		SGA			Main effect AGA vs SGA		Main effect subgroup analysis (AGA vs SGA+ vs SGA-)		AGA vs SGA+	AGA vs SGA-	SGA+ vs SGA-	Polynomial trend analysis
	AGA(n = 18)	SGA+ (n = 18)	SGA- (n = 16)	SGA total(n = 34)	F-value	p-value	F-value	p-value	p-value	p-value	p-value	p-value
Intelligence scores												
WPPSI-R/ WISC-III,	114.7 (15.4)	107.4 (11.9)	101.5 (10.5)	104.7 (11.5)	6.9	.011	5.9	.005	ns	.004	ns	.001
Word/ digit span^a^												
Word/ digit span forwards	4.6 (0.8)	3.7 (0.7)	3.6 (0.8)	3.6 (0.7)	16.3	<.001^c^	8.0	<.001^c^	.004^c^	.003^c^	ns	.001^c^
Word/ digit span backwards	2.8 (1.4)	2.1 (1.1)	1.8 (1.0)	1.9 (1.0)	4.1	.049	2.3	ns	ns	ns	ns	.042^c^
Location learning^b^ ^d^												
Total immediate recall	48.2 (10.4)	46.0 (12.6)	47.6 (9.3)	46.8 (11.0)	0.4	ns	0.3	ns	ns	ns	ns	ns
Proactive interference	4.3 (1.8)	4.7 (2.6)	5.3 (1.9)	4.9 (2.3)	2.3	ns	1.6	ns	ns	ns	ns	ns
Retroactive interference	10.3 (3.1)	10.9 (3.9)	9.6 (2.2)	10.3 (3.2)	2.8	ns	3.2	ns	ns	ns	ns	ns
Delayed recall (30 minutes)	12.1 (2.2)	11.6 (3.0)	9.9 (3.0)	10.8 (3.1)	0.1	ns	4.0	ns	ns	ns	ns	ns
Delayed recognition	13.7 (1.7)	13.3 (2.0)	13.6 (1.3)	13.5 (1.7)	0.1	ns	0.9	ns	ns	ns	ns	ns

Note. data are presented as mean (± standard deviation); AGA = appropriate for gestational age; SGA+ = small for gestational age with postnatal catch up growth; SGA– = small for gestational age without postnatal catch up growth; ns = non significant.

p-value< 0.05 is considered significant, unless specified otherwise

a Main effects after covarying for age

b Main effects after covaring for age and corresponding response times

c Remains significant after covarying for IQ.

d Bonferroni corrected level of significance (α/*n*k) = .01

### fMRI

#### Performance

Table 3 describes performance data of encoding & recognition phase as well as the movement parameters during the scan. Encoding accuracy ranged from 0.6–1.0 for novel pictures and 0.7–1.0 for overlearned pictures, with similar performance in each (sub)group. AGA children responded faster in comparison with SGA children, however this was not statistical significant (F = 3.6, p = 0.06). None of the other parameters described in Table 3 demonstrated significant differences in any subgroup comparison.

**Table 3:** Movement parameters, proportions of accuracy (encoding) and hit rates, false alarm rates, sensitivity (Pr) and response bias (Br) (recognition) for the different stimulus categories in AGA and SGA (combined, SGA+ and SGA-) children (N = 52).

		SGA			Main effect AGA vs SGA*	
	AGA (n = 18)	SGA+ (n = 18)	SGA–(n = 16)	SGA total group (n = 34)	F-value^a^	p-value
Movement parameters^b^						
Total displacement (mm)	0.83 (0.44)	1.16 (1.17)	1.02 (1.06)	1.09 (1.11)	1.23^b^	ns
Scan to scan displacement (mm)	0.20 (0.11)	0.27 (0.20)	0.26 (0.27)	0.27 (0.24)	1.34^b^	ns
Encoding phase						
Proportion correct judgment of novel pictures	0.92 (0.10)	0.88 (0.08)	0.91 (0.11)	0.89 (0.09)	0.1	ns
Proportion correct judgment of overlearned pictures	0.89 (0.12)	0.88 (0.09)	0.90 (0.13)	0.89 (0.11)	0.2	ns
Response time (ms)						
Correct judgment of novel pictures	1260 (228)	1457 (202)	1390 (283)	1425 (242)	3.6	0.06
Correct judgment of overlearned pictures	1199 (270)	1321 (205)	1234 (200)	1280 (204)	0.4	ns
Recognition phase						
Hits	0.66 (0.15)	0.64 (0.19)	0.67 (0.16)	0.66 (0.17)	0.0	ns
False alarms	0.16 (0.14)	0.17 (0.12)	0.16 (0.14)	0.17 (0.13)	1.0	ns
Misses	0.34 90.15)	0.36 90.19)	0.33 (0.16)	0.34 (0.17)	0.1	ns
Correct rejections	0.84 (0.14)	0.83 (0.12)	0.84 (0.15)	0.84 (0.13)	1.0	ns
Sensitivity (Pr)	0.50 (0.22)	0.48 (0.26)	0.52 (0.22)	0.49 (0.23)	1.1	ns
Response bias (Br)	0.25 (0.21)	0.31 90.19)	0.29 (0.20)	0.30 (0.19)	0.0	ns
Response times (ms)						
Hits	1455 (235)	1514 (277)	1495 (217)	1504 (243)	0.0	ns
False alarms	978 (330)	1117 (404)	868 (471)	998 (447)	0.0	ns
Misses	1201 (231)	1250 (229)	1302 9379)	1278 (314)	0.3	ns
Correct rejections	1486 (203)	1492 (187)	1535 (236)	1515 (212)	0.2	ns

Note. Data are presented as mean (± standard deviation) unless specified otherwise; Main effects after covarying for age, p-value< 0.05 is considered significant, p-values between 0.05 and 0.1 are reported. AGA = appropriate for gestational age; SGA+ = small for gestational age with postnatal catch up growth; SGA– = small for gestational age without postnatal catch up growth; Total displacement = overall subject motion relative to the first scan; Scan to scan displacement = scan displacement relative to the previous volume; mm = millimeter; ms = milliseconds; Pr = sensitivity probability; Br = bias response; ns = non significant.

^a^ Analyses of variance unless specified otherwise

^b^ Mann-Whitney U test was applied, median is reported instead of mean and standard deviation

* data on main effect of subgroup analysis and polynomial trend analysis are omitted because no significant differences exist

#### BOLD activation: across group contrasts

**Fig 3:**
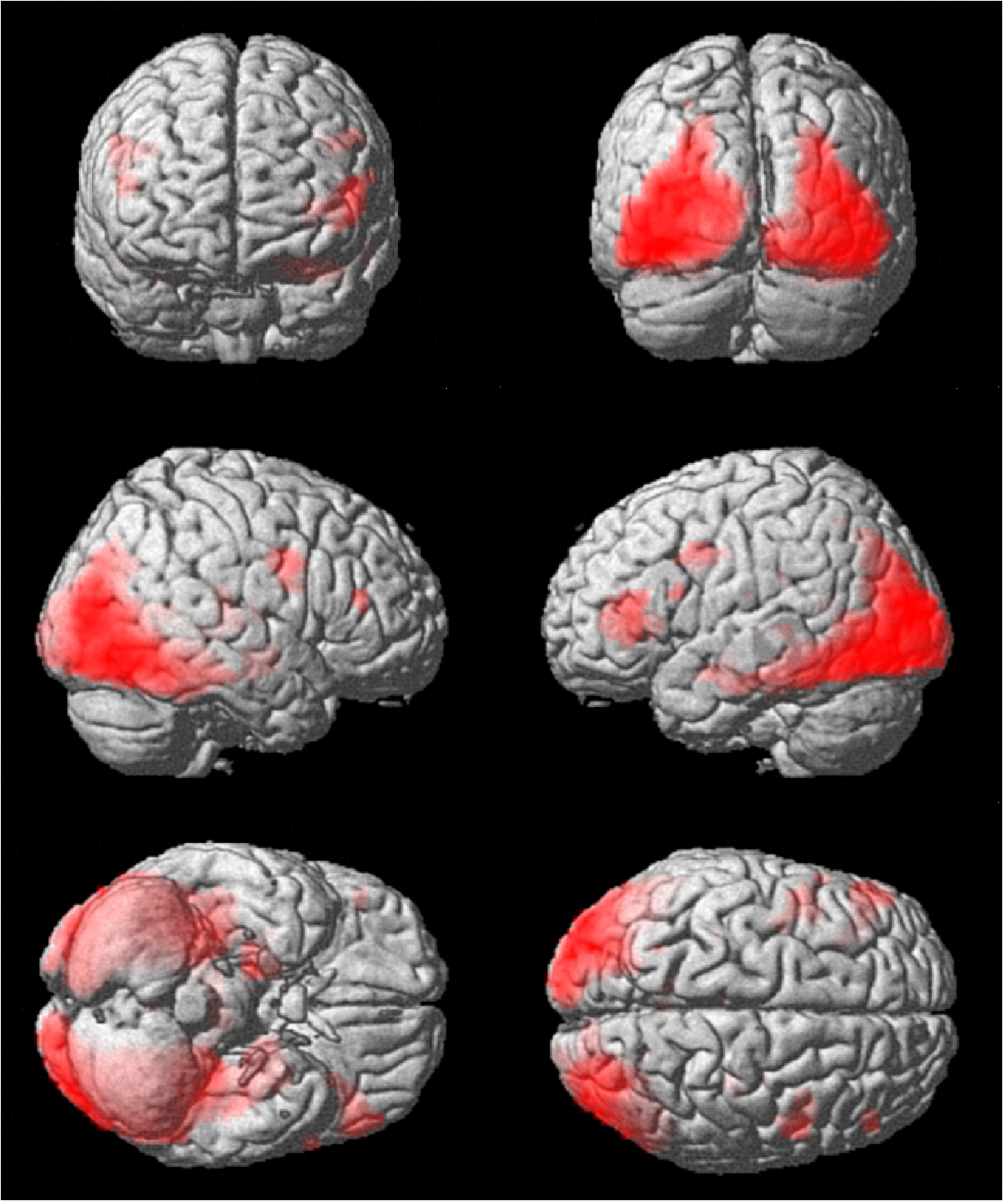
Whole brain activation. Whole brain activation related to encoding novel pictures during the picture encoding task, overlaid on a customized template (Template-O-Matic). Activation is thresholded at p<0.05, FDR-corrected.

**Fig 4:**
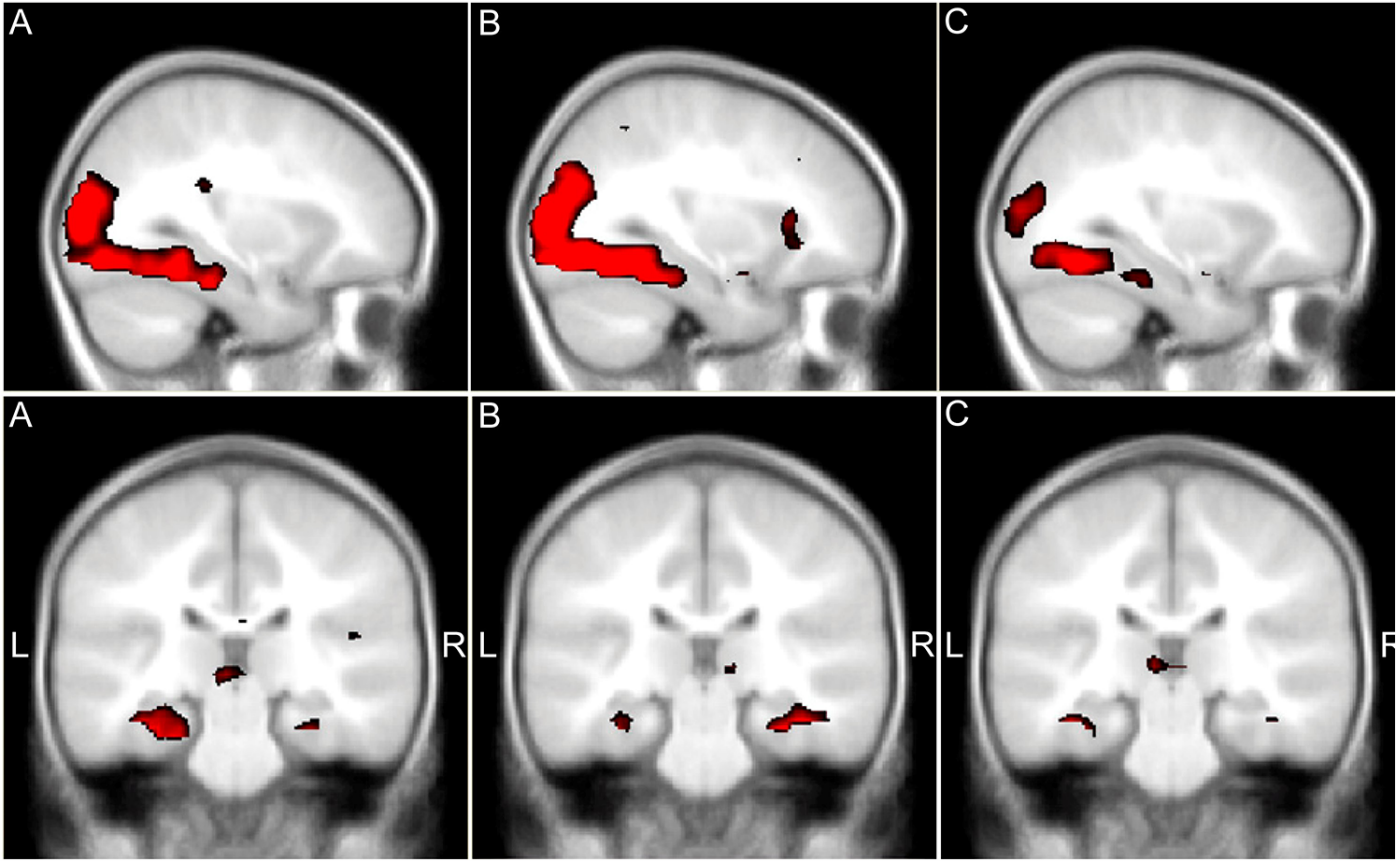
BOLD activation. BOLD activation maps related to encoding novel pictures of the picture encoding task. Upper part: sagittal cross-sections (x = -30, y = -90, z = -9) in AGA (A), SGA+ (B) and SGA- (C) children, representing the left ventral stream with significant activation from the occipital lobe via the fusiform gyrus into the parahippocampal gyrus/hippocampal formation. Functional results are thresholded at p<0.005 for display purposes. Lower part: Coronal cross-sections of the parahippocampal gyrus/hippocampal formation area (x = -21, y = -27, z = -24) in AGA (A), SGA+ (B) and SGA- (C) children. Images thresholded at P<0.005 for display purposes. AGA = average for gestational age, SGA+ = small for gestational age with catch-up growth, SGA- = small for gestational age without catch-up growth.

We analyzed the BOLD signal for the novel vs overlearned contrast in the total sample of children. As expected, whole-brain analysis showed significant increases in fMRI signal of occipitotemporal regions towards the parahippocampal gyrus/hippocampal formation bilaterally, with similar activation patterns in each group (Figs 3 and 4, Table 4). Additional activation was observed bilaterally in the prefrontal gyrus.

**Table 4:** Whole brain analysis; regions of significant increased activation for novel relative to overlearned pictures during the Picture Encoding Test (N = 52).

	Left hemisphere			Right hemisphere		
Contrast	Region of activation	MNI coordinates x;y;z (mm)	Z score	Region of activation	MNI coordinates x;y;z (mm)	Z score
Total sample^a^						
	Occipital lobe	-30;-90;-9	7.55	Occipital lobe	33;-81;15	7.15
	Fusiform gyrus	-36;-60;-12	7.08	Fusiform gyrus	36;-54;-12	7.31
	Fusiform gyrus	-33;-30;-21	6.06	Fusiform gyrus	30;-36;-21	6.08
	Fusiform gyrus	-33;-15;-24	3.96			
	Hippocampus/ Parahippocampal gyrus	-21;-9;-21	4.59	Hippocampus/ Parahippocampal gyrus	21;-9;-12	4.01
	Precentral gyrus	-51;3;39	3.18	Precentral gyrus	51;0;33	3.48
				Transverse temporal gyrus (Heschl's gyrus)	33;-24;15	3.3
	Prefrontal gyrus	-51;39;12	3.58	Prefrontal gyrus	48;33;18	2.96
	Prefrontal gyrus	-42;30;9	3.17			
	Prefrontal gyrus	-60;12;18	2.77			
	Superior parietal lobe	-27;-57;51	2.58			
Group comparison^b^						
AGA > SGA	Parahippocampal gyrus	-21;-27;-24	3.56^c^			
AGA > SGA+	Parahippocampal gyrus	-21;-24;-24	3.13			
AGA > SGA-	Parahippocampal gyrus	-21;-27;-24	3.23			
AGA > SGA+ > SGA-	Parahippocampal gyrus	-21;-27;-24	3.53^d^			

Note. AGA = appropriate for gestational age (n = 18); SGA+ = small for gestational age with postnatal catch up growth (n = 18); SGA– = small for gestational age without postnatal catch up growth (n = 16); MNI = Montreal Neurological Institute template; mm = millimeter; Z score = brain activation difference between novel and overlearned (baseline) condition.

^a^ Regions significant at false discovery rate corrected p <0.05

^b^ Regions significant at uncorrected p < 0.001

^c^ Small volume correction: false discovery rate corrected p = 0.032

^d^ Small volume correction: false discovery rate corrected p = 0.037

#### BOLD activation: group interactions

Comparison between AGA and SGA children revealed lower activation within the left parahippocampal gyrus in SGA children (Table 4). This difference remained significant in both the comparison between AGA and SGA+ and between AGA and SGA- subgroups and persisted after covarying for age. After covarying for IQ, differences remained significant only in comparison between AGA and SGA- (Z:3.06, p = 0.001).

Moreover, there was a linear trend of decreased activation within this area from AGA via SGA+ towards SGA- showing the highest level of activation within the AGA group (Table 4). SGA+ and SGA- children did not differ significantly.

## Discussion

This study is the first functional MRI study in SGA children and the first study using a picture encoding task to investigate the neural foundations of memory in children as young as four years old. Our findings are in line with previous studies in adults and older children demonstrating significant increases in fMRI signal bilaterally in the posterior hippocampal formation and parahippocampal gyrus and in the lingual and fusiform gyri as well as the prefrontal cortex during encoding of novel pictures [7,10,11]. This indicates that this new picture encoding task is suitable for assessing encoding-related activity.

Children born SGA demonstrated lower BOLD activation in a discrete region within the left parahippocampal gyrus compared to AGA children. This difference in BOLD activation was most pronounced in comparison between AGA and SGA- children with SGA+ children showing an intermediate level of parahippocampal activation. Although performance differences were not statistically different, the significantly lower BOLD activation in SGA vs AGA children might indicate impaired parahippocampal functioning during memory encoding. Possibly the fMRI task was not sensitive enough to detect behavioural performance differences, for instance because task duration was limited due to the young age of the population. Alternatively, the lower BOLD activation in SGA vs AGA children might be related to slower response times. The SGA children needed more time for determining whether the presented picture depicted an animal or an object, indicating a lower processing speed. This is in line with a previous study in which SGA children were found to respond slower than AGA children [44]. Our task might not have had enough power to detect these differences on the behavioural level. A third interpretation of lower parahippocampal activation in the absence of memory performance differences in SGA vs AGA children is that SGA children were recruiting less neural resources during memory encoding while maintaining an adequate level of performance during memory retrieval. This could actually mean that SGA children were processing the stimuli more *efficiently* than AGA children, with SGA- children showing the most efficient processing as they showed the lowest parahippocampal activation. Although the observed pattern of results per se supports such an interpretation, we find this explanation less probable in view of the neuropsychological findings in the current as well as other studies [45]. It should be noted that currently there is no general rule as to the interrelation of the magnitude of the BOLD response and task performance. Both stronger and weaker activation have been observed in previous studies in relation to performance. For a more extensive discussion on this topic we refer to Nagel et al. [46] and Heinzel et al. [47].

The group difference in encoding related activity was restricted to the left parahippocampal region, pointing to a specific functional deficit of left parahippocampal gyrus in SGA. However, based on previous findings indicating differences in brain structures [16,17,19] and altered brain network topography [14] in children born SGA, involvement of a larger region was initially assumed. Possibly, ceiling effects in activation of the right parahippocampal gyrus may have concealed group differences in this brain area, as a right hemisphere dominance is often found for encoding of pictographic (as opposed to verbal) stimuli [24]. Another explanation is that the unilateral group difference reflects the predominance of right-handed children in our study population [48]. Due to the low number of left-handed children it was not possible to investigate the subgroup of left handed children separately.

Results of neuropsychological testing indicate lower IQ scores and shorter immediate memory spans in SGA children compared to AGA children. Furthermore and most interestingly, SGA+ children constitute an intermediate between AGA and SGA- children. Thus, although SGA+ children appear similar to AGA children considering their height, catch-up of intelligence and memory seems to be slightly lagging behind. This is in line with previous findings [49–51] and argues for a more intensive follow-up of SGA children who show a postnatal catch-up growth.

Our study demonstrates that, with thorough preparation, task-related fMRI in children as young as 4 years of age is feasible. fMRI in young children is difficult and fMRI studies in children below the age of eight years are scarce [52–57]. The whole appearance of the MRI scanning environment can be intimidating and children may have difficulties in understanding the instructions and requirements of functional imaging tasks. Finally, functional MRI is highly sensitive to movement artefacts. The use of our mock scanner training protocol and extensive coaching of the children resulted in a high percentage of successful scans with adequate task performance (i.e., considerably above chance). Failure rates of the mock scanner session were highest among the youngest children [58].

This study is unique for its population, with children as young as four years old being scanned while performing a picture encoding task. On the other hand, with this population we have set ourselves a rather difficult task and restrictions inherent to this population have to be acknowledged [59,60]. This study was limited by short duration of the fMRI procedure and consequently low number of trials due to limited attention span. Children were excluded when motion exceeded 4mm, which is a liberal threshold in comparison with studies in older children and adults. For functional MRI in general, there are no well-defined or accepted quality criteria for the extent of motion that is still acceptable. In fMRI studies in children, it is not uncommon to include scans with greater motion than 3mm [61–64]. Unfortunately, a considerable proportion of studies do not report clear information on motion characteristics or exclusion criteria [58]. In our study, realignment parameters were similar in each subgroup. To account for individual variability, realignment parameters were modelled as confounders in the general linear model of the individual first-level designs [59]. Another shortcoming of the current study is the relatively high eIQ’s of the AGA children, probably due to selection bias of the healthy control group. Although this might have influenced our results, group interactions between AGA and SGA- were robust against covarying for eIQ’s.

In conclusion, SGA children show lower parahippocampal activation during memory encoding as measured with BOLD fMRI, slower response times as well as subtle neuropsychological disadvantages (including a lower IQ and shorter immediate memory spans). SGA+ children constitute an intermediate between AGA and SGA- children both on the level of brain activation as well as on the level of neuropsychological performance. Results of the current study converge with previous reports, demonstrating that being born SGA poses a child at a higher risk for altered brain function and impaired cognitive outcome.
